# Some Remarks on Maydl’s Operation in Intestinal Obstruction

**Published:** 1896-01

**Authors:** T. D. Wooten

**Affiliations:** Austin, Texas


### TEXAS MEDICAL JOURNAL.

ESTABLISHED JULY, 1885.

PUBLISHED MONTHLY.—SUBSCRIPTION $2.00 A YEAR.

VOL. XI. AUSTIN, JANUARY, 1896. NO. 7.

### Original Contributions.

For Texas Medical Journal.

### SOME REMARKS ON MAYDL'S OPERATION IN INTESTINAL OBSTRUCTION.

BY T. D. WOOTEN, M. D., AUSTIN, TEXAS.

Read at the Meeting of Austin District Medical Society, December 19, 1895.

IN THE few remarks which I have to make on Maydl's operation, I shall confine myself simply to its applicability and technique. This operation is most familiar to the profession as a palliative treatment in carcinomatous stenosis of the colon and inoperable rectal carcinomas; and but a few statements will be necessary to recall to your minds its indications and surgical details.

Of the numerous methods for performing colostomy, described by so many authors since this procedure gained surgical recognition, it would be useless to speak. It is sufficient to say that modern experience has limited the incision to the right or left iliac region, and of these Maydl's inguinal colostomy deserves preference for its simplicity as an operation, and its success as a result.

Omitting a description of the necessary precautions to be taken in all taparotomies, a four-inch incision is made over that portion of the colon to be opened, in the direction of the fibers of the external-oblique. The peritoneum having been divided, the bowel, which usually presents itself at once, is drawn forward until its mesenteric border is on a level with the external incision.

A small slit is then made in the mesecolon close to the gut, and a sterilized pencil, hard rubber cylinder, or better still, a glass rod is passed through it, thus fixing the nuckle of the bowel in the incision, and preventing its return into the abdomen until firm adhesions have formed.

The two limbs of the prolapsed gut are now sewed together below the support by a running Lembert's suture on each side of the flexure. The succeeding steps must depend upon the case in question.

If prompt relief demands an immediate opening of the intestine, the divided parietal peritoneum is drawn forward and stitched to the side of the flexed gut throughout its circumference, then completely closing the peritoneal cavity; the sides of the wound having been protected by a coating of iodoform collodion, the intestine may be opened with best chances against infection.

If, however, the incision of the bowel can be postponed until adhesions have formed, the tedious step of stitching the peritoneum to the intestine may be omitted, as an iodoform dressing will prove an all sufficient protection against outside infection until plastic exudations complete the closure. Whether the intestine is to be cut longitudinally or transversely will, of course, depend upon the temporary or permanent nature of the fistula made; but in cases of doubt the longitudinal one will, I think, most satisfactorily meet any condition that may arise.

The support to the intestinal flexure may be removed about the fourth day, at which time the division of gut may be completed, if a permanent fistula is necessary; or the original opening closed, and the nuckle of the bowel returned to the abdominal cavity, after freeing it from its adhesion.

My special object in speaking of the operation was to advocate its use in certain cases where a temporary opening of the ilium proves a necessary expedient. The objections against the formation of a fistula above the ileo cæcal valve is, I believe, due more to statistics collected without a consideration of the individual cases recorded, than to the special dangers of the operation itself. Enterostomy has been, and will continue to be a procedure of last resort, and it would, therefore, be unreasonable to condemn it simply from numerical data furnished without reference to existing complications. Certain cases of intestinal obstruction will continue to occur where the nature of the obstruction or the enfeebled condition of the patient will prevent any immediate radical procedure. It is in such instances that enterostomy becomes expedient as a means for removing the most pressing symptoms, and affording, after a time, more favorable conditions for radical treatment.

In Nelaton's operation, the one usually adopted now in performing enterostomy, the surface of the gut is simply brought up to the parietal peritoneum and stitched to the margin of the abdominal wound. An incision being made in the center of the united part, and the bowel having emptied itself, the edges of the visceral incision are also stitched to the sides of the wound to insure the patency of the opening.

Now, any one who has attempted to sew the free surface of a gut to the circumference of a two or two and a half inch abdominal incision will fully appreciate the difference between a simple description and a tedious performance. Even without the disturbing influence of respiration, it is difficult, indeed, to hold the gut in position until the sutures are planted, and when completed we have but a single row of Lembert's sutures to meet what must necessarily prove a strain more or less severe.

Again, in emptying the bowel through this fistula, it is impossible to prevent its contents from coming in contact with the wound, and asepsis must depend upon a thorough after sterilization, a process by no means certain, as a complicating peritonitis has already too often proved.

The advantages which Maydl's operation has, when applied to the small intestine, over Nelaton's enterostomy, are simply these:

(1) The gut, in being external, is within complete control, and the stitches can be taken with care and certainty.

(2) The sutures, when made, are not subjected to any undue strain, as the rod-support fixes and holds the intestine in firm position.

(3) When the peritoneal cavity has been closed, and the limbs of the flexure sewed together, the fecal contents may be prevented from coming in contact with the wound by slipping the exposed nuckle through a slit made in a rubber sheeting and turning the patient on the side before opening the intestine.

(4) The operation is applicable through a partially closed median incision where an exploratory laparotomy may have, for any reason, prevented a more radical procedure.

(5) The time for its performance will not, I think, exceed that required by an ordinary enterostomy, and the restoration of the abdominal contents to their normal position, in case this may afterwards be desired, is attended with no more risk to the patient, or difficulty to the surgeon, than would be expected in Nelaton's operation.

Should these few statements, based upon a limited experience, as they are, justify you in applying the principles of Maydl's colostomy, in any case that may demand an enterostomy, I trust that you may at least find my remarks impartial.

